# A multifaceted approach increased staff confidence to develop outside of school hours care as a health promoting setting

**DOI:** 10.1186/s12889-021-12360-w

**Published:** 2021-12-15

**Authors:** Karen Forde, Leesa Costello, Amanda Devine, Ros Sambell, Ruth Wallace

**Affiliations:** 1 School of Medical & Health Science, Edith Cowan University, Joondalup, Australia; 2 Telethon Kids Institute, Nedlands, Australia

**Keywords:** Outside-of-school-hours-care, Health promotion, Confidence, OSHC professionals, Role adequacy and legitimacy, Childhood overweight and obesity, Nutrition and physical activity

## Abstract

**Background:**

Outside-of-school-hours-care (OSHC) services are well positioned to influence the health behaviours of 489, 800 Australian children, and are an important setting for health promotion given the current rates of childhood overweight and obesity and associated health risks. OSHC Professionals are ideally placed to become positive influencers in this setting, although they may require training and support to confidently perform this role. This study piloted a multifaceted intervention strategy to increase OSHC Professional’s confidence and competencies, to support a health promoting OSHC environment with a nutrition and physical activity focus.

**Methods:**

A mixed methods approach was used. Nineteen OSHC Professionals participated in the study, including a face-to-face workshop, supported by a closed Facebook group and website. Role adequacy (self-confidence) and legitimacy (professional responsibility) were measured pre and post workshop and evaluated using non-parametric statistics. Facebook interactions were monitored, and four participants undertook qualitative exit interviews to discuss their experiences with the intervention.

**Results:**

Pre-workshop 68% of participants had not received any OSHC-specific health promotion training. Post-workshop significant improvements in confidence about menu planning, accessing nutrition information, activities and recipes was observed (*P* < 0.05 for all). A significant improvement was observed in role support and role related training (*P* < 0.05). A high level of support and interaction was observed between participants on Facebook and the website was reported a useful repository of information.

**Conclusions:**

Health promotion training, combined with positive social connections, shared learning experiences, and a website improved OSHC Professionals confidence and capacity to provide a health promoting OSHC environment. Health promotion professional development for OSHC professionals should be mandated as a minimum requirement, and such learning opportunities should be scaffolded with support available through social media interactions and website access.

## Background

Overweight and obesity among Australian children is well-known [1] and the World Health Organisation recognises this as a serious global public health issue [2]. In 2017–18, almost one-quarter of Australian children were overweight (17%) or obese (8%), and although these rates have plateaued over the last decade [3], continuing detrimental physical, mental and social effects exist [1] alongside increased risk of obesity as an adult [4]. Obesity related diseases, such as hypertension and type 2 diabetes are increasingly and prematurely diagnosed among young children [1], having significant personal impact on individuals, families and communities, and the healthcare system [5].

There are many social, environmental and policy risk factors contributing to overweight and obesity, apart from individual behaviours such as diet and physical inactivity [1]. Health inequalities and inequities are significant contributors to overweight and obesity in childhood, particularly for those living in low-income households [6]. Therefore, strategies that focus on behavioural change alone, whilst neglecting environmental factors that influence health behaviours, typically have limited, short-term success [7]. Sustainable change must be underpinned by supportive environments and policy, where making the healthy choice is the easy choice [8].

Changing settings to be more supportive of health and healthy choice is “an optimum way to improve population health and health equity” [9]. Creating health promoting environments in settings where children spend most of their time is a positive approach to preventing or reducing overweight and obesity [10] and reducing health inequities [9]. Out-of-School-Hours-Care (OSHC) is one such setting [11], offering unique opportunities because the target demographic (children) is present and the care givers (OSHC Professionals) are ideally situated to facilitate health promoting strategies [11–13].

The Australian Children’s Education and Care Quality Authority (ACECQA) reported that, in 2020, a total of 4608 OSHC services provided care for 489,800 children [12]. Clearly, Australian families engage with OSHC services widely, yet this setting is often underutilised as a health promotion setting [14] and given 17% of Australian children are estimated to be living below the poverty line [15], a missed opportunity to address health inequities [9]. Physical activity and nutritional intake are two key risk factors contributing to overweight and obesity in childhood [1] that can be specifically addressed in the OSHC setting, but for many children, often the period after school is a time when nutrient poor, energy-dense food and beverages are consumed, alongside sedentary activities [16]. To compound the issue, OSHC professionals experience barriers that may impede health promotion strategies, including limited time for food preparation, budgetary constraints, high staff turnover and limited cooking facilities, impacting the nutritional quality of food provided and consumed by children at OSHC [7, 17]. Moreover, OSHC Professionals have limited or no nutrition and physical activity training, impacting their capacity and confidence to provide health-promoting environments [18].

National quality frameworks, guidelines and policies are provided to OSHC services to support their capacity to deliver health promoting environments. Ultimately, these quality frameworks are underpinned by a holistic approach which recognises the physical, social, emotional, and spiritual wellbeing of the ‘whole child’, intended to promote learning opportunities which will sustain positive life habits [7, 19] and educative experiences that promote health equity [20]. OSHC services are responsible for complying with these frameworks and guidelines; and the relevant state or territory regulatory authority are ultimately responsible for monitoring compliance and determining whether OSHC services are achieving National Quality Standards [21]. While this is good news for the Early Childhood Education and Care (ECEC) sector, the authors consider that OSHC services in particular need more support to translate these requirements into every-day practice, underpinning the purpose of this research.

Previous research demonstrates that OSHC-based health promotion programs providing multi-dimensional, settings-based approaches with clear communication and support strategies, are more sustainable than those which seek to change individual behaviours [7, 17]. Tailored programs specific to the needs of a sub-group, i.e., OSHC educators, may result in more equitable outcomes [22]. Furthermore, programs that include staff education and training have successfully boosted perceptions of ‘role legitimacy’ (professional responsibility) and ‘role adequacy‘(self-confidence), leading to increased job satisfaction and motivation, an ideal outcome when implementing a health intervention [17, 23]. Education designed to improve literacy around healthy eating and physical activity, and the provision of supporting resources have also been successful in improving the nutritional quality of food and drinks provided in OSHC settings and the uptake of physical activity in children [7, 24]. There is also evidence that using ‘reimagined’ digital approaches, including social media, to support professional development in education settings is advantageous [25]. Social media can also foster social connectedness and seed learning communities in these settings [25, 26].

The aim of this study was to pilot a three-pronged intervention strategy to understand if it increased the confidence of OSHC Professionals and enhanced their capacity to provide a health promoting environment, focusing on nutrition and physical activity in the OSHC setting. Specifically, we sought to answer the following research questions:

1. Does face-to-face training influence the confidence, role adequacy and legitimacy of OSHC Professionals to provide a health promoting environment?
2. Does a closed Facebook page influence social connections and social learning experiences among OSHC Professionals?
3. Does the provision of online resources influence OSHC Professionals to maximise health promoting opportunities?

## Methods

A mixed methods approach using a triangulation design was utilised to gather qualitative and quantitative data concurrently to answer the research questions [27]. The three-pronged intervention strategy comprised of:

1. face-to-face nutrition and physical activity workshops;
2. an OSHC Professional closed Facebook group and;
3. web-based resources (SNAC_OSHC).

It was theorised that these components would increase OSHC Professional’s confidence and competence to support a health promoting OSHC environment with a nutrition and physical activity focus.

Two theories were used to underpin this study - namely the theory of role adequacy and role legitimacy [23] and the theory of self-efficacy [28]. Role adequacy and legitimacy, collectively known as role perception, is important as it represents job satisfaction and work motivation, key factors driving the success of any health intervention [23]. For SNAC_OSHC to be effective, we theorised that job satisfaction and motivation, and subsequently role perception, should be high. Role support, role experience and role education are components of role perception and foster motivation [23], hence they were an important focus for this study of OSHC professionals.

Self-efficacy was also an integral theoretical underpinning of this study. It is described as an individual’s belief in their ability to perform and achieve a desired outcome and impacts the individual’s confidence, motivation and behaviour [28]. Two of the four sources of self-efficacy - performance accomplishment and vicarious experience – are considered as mechanisms to increase self-efficacy (confidence), and hence, were embedded into competency-based activities during face-to-face workshops, and in shared vicarious experiences on the closed Facebook group associated with this study.

### Intervention (or strategy) development

Initial scoping was conducted with the OSHC sector and an advisory group informed the development of each strategy. As a result, a new section of the existing SNAC website and online community of practice - Supporting Nutrition for Australian Childcare (www.snacwa.com.au) - was developed (SNAC_OSHC) to host and provide online access to resources and support. Permission was obtained from the National Heart Foundation of Australia [29] to repurpose the ‘Eat Smart Play Smart’ (ESPS) Nutrition and Physical Activity manual, which was then hosted on the SNAC_OSHC online portal. This portal was structured according to the core elements of the original ESPS manual. Fact sheets and other supporting resources such as nutrition information and activities, recipes, and links pointing to associated organisations were updated for the OSHC audience in an online capacity. Iterative website development and testing was conducted with the advisory group. A closed Facebook group for OSHC professionals was also established to provide an alternative supportive space to share ideas, ask questions and create positive social connections among intervention participants after the workshop. Given Facebook groups are often considered more immediate and ‘mobile’ [30, 31], the provision of the closed group meant that posts could be ‘linked’ back to resources housed on the main OSHC website.

Two workshops, four hours in length, were conducted on two separate days. At the start of the workshops, participants completed questionnaires designed to collect demographic information and data corresponding to the first research question. This questionnaire was repeated at the conclusion of the workshop session to determine any change in confidence, role adequacy and legitimacy. The workshops unveiled the new OSHC section of the SNAC website and showcased key navigation and registration features. Participants were then involved in various learning activities around recipe and menu planning and the ‘traffic-light’ system [32]. All workshop participants were provided with a ‘take home’ folder containing key resources to share with other staff at their service. At the conclusion of the workshops, an activity involving post-it notes enabled participants to express ideas - as an inclusive warm-up activity prior to a short focus group to further explore the usefulness of the workshop content and the barriers and enablers to implementing change. Participants were also encouraged to sign up to the closed Facebook group during these workshop sessions so that they could seek support from other attendees as needed. Seven weeks post-workshop, semi-structured telephone interviews were conducted to determine participant confidence to provide a health-promoting environment, any changes made at the service, and to gather feedback about the elements of the intervention – workshops, website and Facebook group.

While the theoretical underpinnings of this study have been described earlier, it is important to acknowledge projects delivering intervention-type strategies should utilise planning or guiding frameworks to support the management of the project itself. Hence, the 5 M Model was selected as a guiding framework to steer the implementation of the intervention described above [33]. It was developed specifically for the purposes of maximising interventions in the OSHC setting and was based on the strength of systematic reviews, policy documents and literature regarding OSHC competencies. Rather than a more traditional knowledge-based approach, the 5 M Model provides a competency-based model that can be used to augment successful implementation of programs into the OSHC setting [33], and thus was considered ideal for this study. The 5 M’s - Mission, Motivate, Manage, Monitor and Maximise - were integrated into this study as tabulated below (Table 1).

**Table 1:** Integrating the 5 M Model and SNAC_OSHC

5 M Component	Workshops	Facebook group	Website
Mission	A clear purpose was developed to support competency development and subsequently confidence: SNAC_OSHC is *committed to assisting and supporting OSHC Professionals in developing and providing a health promoting setting for all children.*		
Motivate	Facilitated discussions about the role of OSHC Professionals as healthy influencers, growing confidence; Workshop manuals provided supporting resources to ensure competency	OSHC Professionals were motivated through collaboration, competitions and idea-sharing; Vicarious experiences nurtured confidence and grew competency	Supporting resources were provided so OSHC Professionals had confidence in the messages being conveyed and were competent at delivering them.
Manage	Practical demonstrations to increase health promotion competencies	Reinforcing competencies through conversations about credible resources, which also supported confidence building	Practical tools, e.g., safe food handling, policy guidelines, menu-planning resources, recipes. Grew OSHC Professionals confidence in their competence to embed health promoting practices
Monitor	Included strategies to monitor children’s food intake and their involvement in activities to foster inclusivity, thus building confidence to do so. Discussions about ways to encourage healthy behaviours, e.g., leading by example, giving children input into menus and activities, praise for participating, supporting competency in these tasks.	Facilitated conversations about successful strategies outside of the workshop environment built OSHC Professionals confidence and provided practical examples to increase competence.	Supporting resources provided As above
Maximise	The sum of the three intervention strategies were intended to have a greater impact on OSHC Professional confidence and competence than one individual intervention strategy, with the intent of maximising the effectiveness of the intervention and increasing the likelihood of achieving the study aim.		

Derived from Weaver et al., (2012) [33]

### Recruitment

Sampling was both purposive with participants recruited from a large Perth-based OSHC organisation and convenience via advertisements placed on social media. For inclusion in the study, prospective participants needed to be employed as an OSHC Professional, residing in the Perth metropolitan area; and available to attend a free 4-h workshop. Participants completed the workshop as Professional Development training within working hours and were remunerated by their employer. OSHC Professionals who could not attend the workshop were invited to access the website and closed Facebook group.

This pilot study aimed to recruit as many participants as possible to engage with the OSHC program and provide insightful data to inform the research questions. All participants were offered equal access to all elements of the SNAC-OSHC program i.e., no specific intervention and control groups were needed. Pilot studies are commonly set up this way, as an efficient way to collect initial data with limited timeframes and resources [34].

### Data collection

Given the mixed method study design, data were collected using several different methods at several time points in the study (Table 2).

**Table 2:** Overview of data collection methods

Strategy	Data collection method	Variable measured
**Face-to-face workshops**	***Pre-workshop questionnaire*** *(****quantitative****)* *(10 questions) -* Based on: Wallace et al. (2015); Skinner et al. (2009) Administered on paper, collated in Qualtrics	• Demographics; • OSHC service facilities available; • History of health promotion-based training; • Confidence levels (Likert scale); • Role adequacy and legitimacy (Likert scale)
	***Workshop activities (qualitative)*** ‘*Have a voice activity’* – participants wrote their responses to three questions on post-it notes and attached them to the relevant question board. Followed by an *informal focus group* discussion (recorded with permission), providing participants the opportunity to further explore emerging themes.	• Workshop feedback • Intent to implement change • Enablers and barriers to implementing change
	***Post-workshop questionnaire*** *(quantitative)* *(19 questions) -* Repeat of pre-workshop questionnaire and additional questions: • Relating to the workshop as an indicator of role adequacy (3 questions yes/no responses) (Skinner et al., 2009) and workshop evaluation (6 questions – Likert scale). Administered on paper, collated in Qualtrics	In addition to the variables measured in the pre-workshop questionnaire, further exploration of role adequacy and legitimacy, and workshop feedback
	***Exit interviews (qualitative)*** 7- weeks post-workshop, semi-structured telephone interviews of 20–30 min duration (recorded with permission), comprising of 22 discussion points. Participants were also asked to provide one word to summarise their overall experience with the intervention.	• Confidence to provide a health promoting environment • Changes made at service • Feedback about intervention elements – workshop, website and Facebook group
**Website**	*Google analytics (quantitative)* Measured website traffic, membership numbers, page views, resources downloaded	Website use
**Facebook group**	*Facebook analytics (quantitative and qualitative)* Closed Facebook group established at time of workshops – monitored for 7 weeks Page and post likes, shares, comments and member interactions.	Facebook group use Insights into how the intervention resources were being used and the impact they were having

### Quantitative data analysis

Pre and post workshop questionnaires were entered into Qualtrics [35] and data were exported to SPSS [36]. Wilcoxon signed rank tests were performed to measure the effect of the intervention on confidence levels, and role adequacy and legitimacy indicators.

Google analytics and website downloads provided data about new SNAC_OSHC members, page visits, and resource downloads from the SNAC_OSHC portal. Facebook analytics provided data relating to the number of posts made, viewed or liked by members.

### Qualitative data analysis

Data from the workshops were analysed qualitatively in two components: The first involved the tabulation of the post-it note activity to identify common themes arising from the responses provided during this activity. The second component involved the analysis of the transcripts generated from the focus group sessions. Both sets of data were entered into NVivo [37] along with the qualitative data collected from the Facebook group and from the exit interviews. Data were coded by the first author initially and then checked by two of the other authors for interpretation consistency before second and third level coding was conducted. This inter-coder reliability approach [38] provides the methodological rigour to construct a thematic representation of the qualitative insights collected in this study. Combining qualitative and quantitative data collection methods allowed the researcher to examine and interpret the interrelationship of themes [39], a triangulation approach common to mixed methods studies [40], that helps to corroborate and validate themes and findings [41, 42] and provide rigour to mixed methods research [43].

## Results

The results are presented to correspond with the data collection methods used. Demographic data is presented first below.

Nineteen participants were mostly employed in OSHC service setting and most were female, almost three-quarters aged between 18 and 40 years, half had a TAFE qualification and had worked in the industry for 1–5 years (Table 3). Two-thirds of participants (68.4%, *n* = 13) had not received any food and nutrition or health promotion training previously.

**Table 3:** Demographic information of participants

		Number	Percent
**Gender**	Male	2	10.5
	Female	17	89.5
**Age (years)**	18–30	11	57.9
	31–40	3	15.8
	41–50	4	21.0
	51 and over	1	5.3
**Highest**	High School	5	15.8
**Education Level**	TAFE	10	52.6
**Achieved**	University Degree	6	31.6
**Time in OSHC**	< 12 months	8	26.3
	1–5 years	9	47.4
	> 5 years	5	26.3

All participants employed in an OSHC service setting (*n* = 17) had access to a variety of food preparation equipment, including a fridge and toaster; most had access to an oven (*n* = 15), 9 had a blender, and only 4 had access to an electric fry pan. All participants (*n* = 19) reported having a food and nutrition policy and a weekly menu planner at their service. Most participants (n = 15) reported a weekly food budget that ranged from $120 to $249 for between 100 and 200 children per day, equating to, on average, approximately $1.20 per day/child.

### Pre- and post-workshop questionnaires

Most participants had *confidence* to perform a range of tasks related to planning menus and physical activities, accessing nutritious food ideas and nutrition-based activities prior to the intervention (Table 4). Post workshop confidence had significantly increased in four areas relating to nutrition and health promotion skills and behaviours (*P* < 0.05 for all, Table 4). The effect size was large for 80% of questions suggesting that the workshop and learning experience contributed to growth in confidence. There was no significant improvement in role modelling, as at baseline most participants already considered themselves to be ‘very confident’.

**Table 4:** Pre- and post-workshop self-reported confidence levels for OSHC health promoting aspects

How confident you feel to perform the tasks below?		Very confident	Confident	Somewhat confident	Not at all confident	***p***-Value	Effect Size^a^
I can plan a nutritious weekly menu	Pre	3 (16.7%)	12 (66.7%)	3 (16.7%)	0 (0%)	0.004	0.688
	Post	13 (72.2%)	5 (27.8%)	0 (0%)	0 (0%)		
I can access nutritious food ideas for children	Pre	5 (27.8%)	9 (50%)	3 (16.7%)	1 (5.6%)	0.003	0.694
	Post	16 (88.9%)	2 (11.1%)	0 (0%)	0 (0%)		
I can access a variety of nutrition-based activities	Pre	5 (27.8%)	9 (50%)	3 (16.7%)	1 (5.6%)	0.003	0.694
	Post	16 (88.9%)	2 (11.1%)	0 (0%)	0 (0%)		
I can plan physical activities for children	Pre	8 (44.4%)	9 (50%)	1 (5.6%)	0 (0%)	0.013	0.588
	Post	16 (88.9%)	2 (11.1%)	0 (0%)	0 (0%)		
I role model healthy behaviour to children	Pre	12 (66.7%)	6 (33.3%)	0 (0%)	0 (0%)	0.102	0.385
	Post	16 (88.9%)	2 (11.1%)	0 (0%)	0 (0%)		

Wilcoxon Signed Rank Test, ^a^Effect sizes of 0.1, 0.3 and 0.5 are classified as small, medium and large, respectively

Participants agreed with *role adequacy* and *role legitimacy* statements at baseline. Post-workshop, significant improvements in role adequacy and support, level of experience and training was reported (*P* < 0.05 for all, Table 4). A large effect size was seen for questions around role adequacy (*p* = 0.021, effect size 0.544), role support (*p* = 0.014, effect size 0.577) and level of training (*p* = 0.013, effect size 0.586), indicating that confidence had grown in these areas post-workshop.

The majority (94.4%) of participants reported the workshop as excellent, the content as useful, relevant, and meeting their expectations. Of the workshop content, the topic “OSHC as a Health promoting setting” and “Nutrition and physical activity” were ranked the highest by 88.9% of participants (*n* = 16). Additional topics suggested for future workshops included: gluten and lactose free recipes and information about standard serve guides.

### Workshop activities

Qualitative data collected during the post-it note activity revealed that participants valued the content delivered in the workshop because it seeded their own ideas about how they could improve their menu planning.

“*Doing the food preparation activity [during the workshop] has given me lots of ideas, and the recipes in this folder and on the website [SNAC_OSHC] will make things so much easier…you know they are going to be healthy…I can’t wait to try them at my service”* (Participant 4).

Participants described how listening to others share ideas was particularly helpful. Participants appreciated the tools and resources provided to them in hardcopy form and commenting in particular, how useful the traffic light system was, noting how it helped them to learn how to “add fruit and veggies into snacks to make them better” (Participant 5) and expressing they would like to implement this at their service.

*“[The traffic-light system] would make planning so easy because if its red, it’s off the list and if it’s amber we can get it over to green by adding other things to it…so simple”* (Participant 3).

Participants also expressed positive sentiments about the website resources, especially being able to access to it whenever and wherever they needed.

In the focus group activity which followed, participants expanded on their initial thoughts, with discussions centred on the barriers to implementing their health promoting intentions. Table 5 highlights the coding process where indicative quotes are presented to demonstrate how themes were derived using data from the post-it note activity and focus groups.

**Table 5:** Themes derived from the coding process

Theme	Indicative Quotes
A consistent approach to professional development	“*sometimes it is just the co-ordinators getting the training and [they] have to communicate it back to the assistants and if they [the assistants] could access it themselves, they would have a better understanding of it all”* (Participant 5). *“getting colleagues support and involved”*^a^
Definition of team roles	[we need] “*team planning to ensure roles and ideas are sorted*”^a^
Limited budgets	*“it is 75c per child per day…. if you had …. 35 [children] across the day ….times 5 [days]…. add on the delivery fee on top of that …. [the] budget would be about $130 per week plus $13 delivery….”* (Participant 1).
Lack of policy awareness or involvement in policy development	[I would like] “*to help head office write policy”*^a^*.*
Children’s allergies, intolerances and food preferences	A summary of participants thoughts (not direct quotes)^a^: This was noted as a key element required in the professional development so OSHC Professionals can respond appropriately to children’s likes/dislikes within the capacity of the available resources.

^a^Quotes taken from post-it notes so unidentifiable

### SNAC_OSHC closed Facebook Group

At the conclusion of the workshops, 17 participants joined the Facebook group and promoted it by referring others and sharing the group with their networks. Within two months there were 113 OSHC members. Participants’ use of the Facebook group was minimal during the first weeks of the study with 66% (*n* = 12) of posts being made by the researcher. Initial researcher-driven posts that aimed to prompt discussion (likes and shares) about recipes and snacks had little impact on interaction. However, when participants were asked to respond to a simple poll about the number of children attending their service, there was a sharp increase in activity. This was leveraged by the researcher adding new posts designed to encourage participants to share ideas about indoor games that promote physical activity. Seven participants responded to the post and began to interact in a positive way, exchanging pleasantries and sharing experiences. Another participant shared a photo of frozen yoghurt drops as an after-school snack idea, and over the following few days other participants shared their own variations of this idea. At this point, the closed Facebook group was showing positive signs of becoming self-sustaining, particularly given participant-led posts began to overtake the number of researcher-led posts. By the end of the study, researcher-led posts accounted for 48% (*n* = 24), and 52% (*n* = 26) of posts were made by participants.

Participants’ Facebook involvement ranged from highly active, demonstrating engagement by posting, commenting and liking other posts, through to observation only. Observational participation was confirmed as Facebook analytics identified the number of group members who had ‘seen’ a post. The cross-section of different ‘user’ types was comparable to other health-focused, social networks and is described the “90–9-1 rule” [44]. This rule asserts that ‘lurkers’ (90%) observe but do not contribute; ‘contributors’ (9%) engage infrequently; and ‘superusers’ (1%) create most group content [44], which aptly summarises the usage culture of the SNAC_OSHC closed Facebook group. Most SNAC_OSHC Facebook members were considered ‘lurkers’, as they read posts but did not like, comment or post themselves.

During the four exit interviews participants described their interactions in the closed Facebook group in positive terms, motivating them to try out some of the ideas being posted and shared. They described value in ‘seeing’ what other professionals were doing in other OSHC services without having to physically visit them. The nature of the Facebook posts also revealed a sense of support and appreciation of idea sharing in particular. For example:

“*The FB page made my assistant and I discuss what we were going to put up, which made us think about new things to do at our service. We were tagging each other in posts to check out. We don’t get to visit other OSHC’s so it is so good to be able to connect and see what are others are doing. Not just other people from my organisation, but other people from other OSHC’s too. It is really friendly. Excellent idea sharing things. Very positive experience. Really positive. Lots of sharing ideas and networking. Wish more people from my organisation participated…*” (Participant 3).

“*I liked the Facebook page. I ‘liked’ and ‘commented’. I actually became friends with some of the people at the workshop and we snapchat ideas to each other too. It is the new way to network. It was a positive experience*” (Participant 1).

“*Very positive, even though didn’t post, always checked updates and page for new ideas. Was great*” (Participant 6).

These types of interactions seemed to seed the beginnings of important new networks and potential friendships with other OSHC professionals. They did, however, describe a desire to see more people joining and posting ideas. Two of the interviewees recommended the Facebook group to colleagues at their service, while one pointed out that most of the colleagues from her service were ‘old school’ and did not use social media.

### Website analytics

**Fig. 1:**
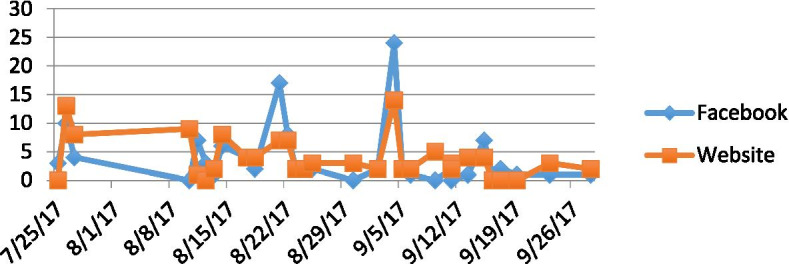
SNAC_OSHC Facebook Group and Website Members Growth

All 19 workshop participants registered for the website. Through referral and sharing of the web page details, within 2 months, membership had increased by 144 new OSHC members. This study did not set out to grow a community outside of the study participants, however, the natural evolution of members on both the Facebook group and website demonstrated an emerging online SNAC_OSHC community (Fig. 1).

Website metadata provided data about the traffic to the top ten pages visited on the SNAC_OSHC website. The most visited page was the ‘No Cooking – Recipe’ page with 228 logged visits. This page is the access point for all non-cooking recipes. The Toolbox-Nutrition page, which houses nutrition-based learning activities, ranked as the second most frequented page (209 visits).

The exit interview participants noted the website was very easy to use and they particularly liked the recipe and activity pages. These participants also described how making recipes accessed from the website had increased the children’s participation in preparing and trying new foods. However, they also requested that more recipes be made available via the Facebook group and noted that healthy treat recipes were in high demand there.

### Exit interviews

Participants described their interactions with the website and closed SNAC_OSHC Facebook group, demonstrating how engaged they were with these aspects of the intervention:

“*I was on it every day…every time a notification popped up, I checked it out …. it’s good to see what others are doing and try it ourselves. We chat all the time about what we are going to post, it’s been really positive for us*” (Participant 5).

*“I have been using it [Facebook] a lot lately. It is really easy to share your ideas and help others…I get ideas from what others put up too”* (Participant 3).

The exit interviews also revealed the changes made at participants OSHC services since they attended the workshops. They described seeing an increase in children’s consumption of fruit and vegetables at their services. For example, one participant noted that they were focused on serving more vegetables and encouraged children to try something different everyday (Participant 5). They also made the following comments about the workshops

*“the food demonstration at the workshop helped me to think and be more creative with the way I present the same food… like capsicum… I use it as a train now” (Participant 1)*

*“increased awareness of better menu planning, the traffic light system opened my eyes to better choices” (Participant 6)*

These same participants talked about the type of changes they would still like to make at their services. For example, more variety in their menus, a wider range of recipes, changes to the physical layout of their rooms, seeking larger budgets, and more feedback from management.

*“The training was really beneficial. I went straight into work and hung the new posters… the pack [workshop folder], was great! It was really good my assistant came too and I didn’t have to relay anything!” (Participant 5)*

*“The folder was such a good resource and has helped me a lot. Love networking with others and seeing what everyone is doing, and sharing ideas” (Participant 3)*

*“Was really good, helpful, much needed. New co-ordinators would really benefit from doing this before starting work in OSHC… Actually even childcare workers outside of OSHC would benefit from this I think…”(Participant 6)*

While such changes may take more time, they did seem to be, at least in part, due to their participation in the study, which participants seemed to enjoy. The comments above highlight these sentiments and summarise the collective benefits of the SNAC_OSHC intervention.

## Discussion

This study aimed to explore how a three-pronged intervention strategy might influence the confidence of OSHC Professionals and enhance their capacity to provide a health promoting environment, focusing on nutrition and physical activity in the OSHC setting. The results were presented earlier in the paper to correspond with the data collection methods used. This data has been synthesised and presented in the proceeding discussion section under headings that relate specifically to the research questions.

### Does face-to-face training influence the confidence, role adequacy and legitimacy of OSHC professionals to provide a health promoting environment?

OSHC Professionals self-reported confidence levels increased significantly across four health promotion areas (Table 4) consistent with other OSHC specific intervention approaches [17]. Prior to the workshop, only 21% of participants reported being ‘very confident’ about planning a nutritious weekly menu, however, post-workshop this had increased to 72.2%. This increase may be attributed to introducing the traffic-light system, which appealed to many participants. The traffic-light system was a ‘hot topic’ during the reflective focus groups, with most participants (*n* = 18) unaware it existed but willing to implement it at their service, describing it as user-friendly and easy to follow, as described in other WA research [45]. The lack of participant awareness about the traffic-light system is concerning, given it is embedded in all WA schools [46] and many OSHC services are located on school premises [32]. The implementation of a similar system at OSHC services could create a clear mission as suggested in the 5 M model, assist in building confidence around menu planning and nutritious food provision, whilst increasing role motivation to support children to eat healthier food [23, 33]. In our findings, the traffic-light system also seemed to increase participants confidence to access health promotion activities. For example, one participant modified the system to create an activity and shared it with the Facebook group, implying high confidence and motivation levels.

In relation to menu planning and food ordering, inadequate food budgets were highlighted as a common concern, which has been reported elsewhere [11], and can impact the quality and type of foods provided [16, 47]. During the workshop, participants discussed ways of maximising food budgets whilst maintaining healthy food choices, concluding that their confidence to provide nutritious food on a limited budget had increased through sharing practical strategies.

Confidence to source nutritious food ideas and recipes increased from 31.6% pre-workshop to 88.9% (Table 4) post-workshop, and the focus group discussions reinforced this increased confidence with many participants reporting using the workshop and website recipes, finding them useful and easy to implement. Pre-workshop, participants sourced recipes from various websites but were unclear about their quality and where they could seek out more reliable information, a seemingly common experience in the Early Childhood Education and Care setting, as reported elsewhere [48, 49]. In our study, attendance at the workshop increased their confidence to source reliable recipes and resources, which was supported by access to the SNAC_OSHC website and closed Facebook group.

Prior to the face-to-face workshops, most participants had not received any health promotion training, a common phenomenon across the OSHC industry [17]. This study recognised that role adequacy is influenced by certain key requirements: role-related training and the perceived usefulness of this training [23], vital to build OSHC professionals’ confidence. Our study found that role adequacy (confidence) and role legitimacy improved significantly (Table 6) following the face-to-face workshops, in terms of role support, confidence, adequacy and training. This study set out to influence OSHC professional’s confidence, so it is not an unexpected result, but highlights the need for ongoing OSHC professional development to build confidence and effectively maximise health promoting opportunities in the OSHC setting.

**Table 6:** Role Adequacy and Role Legitimacy; Pre and Post Workshop

Professional development in your OSHC role		Strongly Agree	Agree	Disagree	Strongly Disagree	***p***-Value	Effect Size
*Job satisfaction*	Pre	13 (72.2%)	5 (27.8%)	0 (0%)	0 (0%)	0.317	−0.236
My experience in OSHC has been rewarding	Post	12 (66.7%)	6 (33.3%)	0 (0%)	0 (0%)		
*Work motivation*	Pre	17 (94.4%)	1 (5.6%)	0 (0%)	0 (0%)	0.564	−0.136
I believe addressing nutrition and health promoting areas in OSHC is important	Post	16 (88.9%)	2 (11.1%)	0 (0%)	0 (0%)		
*Role adequacy*	Pre	8 (44.4%)	9 (50%)	1 (5.6%)	0 (0%)	0.021	0.544
I am confident in my ability to address nutrition and health promoting areas in the OSHC setting that I work	Post	15 (83.3%)	3 (16.7%)	0 (0%)	0 (0%)		
*Role legitimacy*	Pre	16 (88.9%)	2 (11.1%)	0 (0%)	0 (0%)	1.000	0.000
I have a responsibility to promote a healthy OSHC environment	Post	16 (88.9%)	2 (11.1%)	0 (0%)	0 (0%)		
*Role support*	Pre	10 (55.6%)	8 (44.4%)	0 (0%)	0 (0%)	0.014	0.577
If I needed to, I could easily find a workplace colleague for support in OSHC nutrition and health promoting	Post	16 (88.9%)	2 (11.1%)	0 (0%)	0 (0%)		
*Level of experience*	Pre	6 (33.3%)	10 (55.6%)	2 (11.1%)	0 (0%)	0.035	0.498
I believe I have sufficient experience in nutrition and health promoting areas for OSHC purposes.	Post	11 (61.1%)	7 (38.9%)	0 (0%)	0 (0%)		
*Level of training*	Pre	5 (27.8%)	10 (55.6%)	2 (16.7%)	0 (0%)	0.013	0.586
I believe I have sufficient training in nutrition and health promoting areas for OSHC purposes.	Post	12(66.7%)	6 (33.3%)	0 (0%)	0 (0%)		

Wilcoxon Signed Rank Test, *Effect sizes of 0.1, 0.3 and 0.5 are classified as small, medium and large, respectively

OSHC services are responsible for complying with national quality frameworks, guidelines and policies to deliver health promoting environments that recognise the wellbeing of the ‘whole child’, promote learning opportunities to sustain positive life habits and negate health inequities. The SNAC_OSHC program – tailored specifically for the needs of a sub-group (OSHC Professionals) among a broader population (the Early Childhood Education & Care sector) [50] – has demonstrated an increase in OSHC Professional confidence and competence to comply with ‘up-stream’ strategies, i.e., frameworks, guidelines and policies that have a greater impact on reducing health inequities [50], enabling them to maximise health promotion opportunities for children, especially beneficial for those experiencing health inequities.

### Does a closed Facebook page influence social connections and social learning experiences among OSHC professionals?

Similar to other health-focused, online social networks, participants interacted with the closed Facebook group at varying levels. These range from a small number of ‘super-users’ (1%), ‘contributors’ (9%), as well as ‘lurkers’ (90%), who make up the majority. This is often referred to as the ‘cybercultural phenomenon’: the 90–9-1 rule [44]. Despite the low number of ‘superusers’ emerging from the group, members testified how, almost instantaneously, a supportive space was created and experienced by members who logged in regularly to read posts and follow links.

It could be that the rapid responses to posts and comments participants received, were motivated by the experience of an ‘ego boost’, where members are ‘seen and rewarded’ for their participation [51]. This type of reinforcement is often vital to confidence building and raising levels of role adequacy [23]. The Facebook analytics suggest that all members were reading the posts, despite them being generated by the few superusers who created them. Hence, their level of engagement indicates that they were able to find support in this environment, given many participants indicated that support from their managers ‘on the ground’ was lacking. In this case, the Facebook group was instrumental in providing sustained support after the workshops concluded. Weaver et al. [11] specifically discussed this concept as an important ‘reinforcement’ technique to ensure the success of more traditional education settings such as workshops. Enhancing intervention implementation, in this case through ongoing support, is also represented in the 5 M model [33] as a way to maximise the effectiveness of the intervention overall. In previous research which was conducted in relation to the SNAC website, providing support through positive connections and interactions (along with reliable information) was pivotal to the evolution of the SNAC community [52]; one which has ultimately paved the way for this research into the OSCH sector.

The SNAC_OSHC closed Facebook group had the characteristics of a developing community-of-practice – a powerful tool to facilitate informal learning [53], and one which brought about increased knowledge and peer support among the OSHC professionals accessing the group. Participant posts and interactions highlighted the value placed on idea sharing, which created interest and momentum among members. Building community of practice in this way can be key to organisational success by providing a social structure that facilitates sharing – of information, experiences, tips and ideas [53], building a deeper understanding of industry knowledge through the shared experiences of others [52] and building confidence through vicarious experiences [28]. Such changes in the OSHC setting may also be more supportive of health and healthy choices – providing an optimal pathway to improving the health of OSHC professionals and the children they educate, which may help to reduce health inequities experienced by some of these children [9].

### Does the provision of online resources influence OSHC professionals to maximise health promoting opportunity?

The SNAC_OSHC website provided a repository of credible health-promoting resources for OSHC professionals to support their role. Such online repositories are growing in popularity as they are available 24/7, providing appropriate resources in one convenient space [25, 54]. Such a suite of resources relates to the ‘manage’ element of the 5 M model, as it provides practical tools that can help reinforce competencies [33]. In particular, the ‘no-cooking – recipe page’ was one of the most visited areas (228 visits) of the SNAC_OSHC website; the access point for all non-cooking recipes and reflective of the limited food preparation facilities most OSHC Professionals experience.

Website metadata confirmed a regular flow of traffic and participants reported it as a useful tool that assisted with menu planning and activities, providing trustworthy information. Other authors have reported that when professionals are confident about the credibility of resources and can access them easily, they are more likely to reuse the repository e.g., Maloney et al., 2013 [54]. SNAC_OSHC participants reported returning to the website regularly to access resources and recommended it to other colleagues and parents. Web analytics and participant feedback demonstrated the online resources added value and ongoing support to providing a health promoting environment, much like the original SNAC study [52]. Similar to the results from a US study about the online preferences of early years educators, our results also support the notion that Australian early years’ educators are receptive to online materials and are happy to embrace them in their working lives [55].

Although the website analytics, compared to the Facebook analytics, suggest that the website was more popular, some participants requested that more recipes, especially healthy treat recipes, be made available through the closed Facebook page. The popularity and usefulness of social media sites, such as Facebook, to foster social connectedness and seed learning communities, has been reported as an important setting for future health promoting strategies [56, 57]. The rapid uptake of and engagement with the SNAC_OSH resources demonstrate a need for contemporary and user-friendly support in the OSHC sector, which can be provided through online interventions like this.

### Strengths

The strengths of this study are that it builds on a substantial body of work relating to the SNAC community of practice and extends this support to the under-supported OSHC sector. In the early stages of the study, there was significant input from the OSHC industry which facilitated recruitment, ensuring it was relevant and guided by industry collaborators. The 5 M model provided a framework that augmented the successful piloting of this program, and the provision of the three strategies (workshops, Facebook and SNAC website) provided a comprehensive suite of resources designed to maximise impact.

### Limitations

It is possible that some of the responses provided by participants were subject to social desirability given the researcher formed relationships with participants, initially during the face-to-face workshops, and later, during the Facebook interactions. While this may be a reasonable assumption in quantitative philosophy and has been considered here, building relationships with participants is considered a primary strength in qualitative research [58]. Other limitations may also arise from the smaller than expected sample size, which may have impacted the effect size of the confidence levels. Hence, the self-reported increased confidence may be overstated. In recognising this, the small sample size is not considered a limitation from a qualitative perspective, particularly given the rigorous process involved in coding and member checking [38]. It is worth considering, however, that participants were paid for their time to attend the workshops, although attendance was voluntary. Therefore, those participants who *did* attend were, perhaps, more interested in health promoting topics and professional development in this regard. If this was the case, the findings need to be understood with this group in mind and may not reflect broader views shared by others in the OSHC workforce.

## Conclusions and recommendations

This pilot study used a multifaceted approach to develop OSHC as a health promoting setting. A three-pronged strategy enhanced the confidence of OSHC professionals and assisted them to implement health promoting environments, fostering and creating a supportive environment for the children attending these services.

A holistic, multifaceted approach brought about improvements in the confidence of OSHC professionals to support health promoting environments, with the social media aspect resulting in a more engaged and connected workforce. A larger, longitudinal study is required to explore the sustainability of similar strategies and determine what is required to maintain confidence and motivation in the longer term. This study suggests that a multifaceted approach can maximise benefits to participants and increase the likelihood of intervention success. Essentially, this study contributes to our ultimate raison d’etre: to improve the capacity of OHS professionals to deliver health promoting environments that support the health and wellbeing of Australian children and contribute to efforts aiming to reduce overweight and obesity in childhood.

To increase the confidence of OSHC Professionals to support health promoting environments, the following recommendations are suggested:

1. Introduce health promotion professional development for OSHC professionals as a minimum requirement, and scaffold this learning with support available through Facebook interactions and website access.
2. Introduce a modified version of the traffic light system as standard practice in OSHC settings, through food and nutrition policies at local OSHC settings, State and National levels. OSHC professionals found this system user-friendly and were keen to adopt it at their services.
3. Promote the use of social media platforms to engage OSHC professionals and provide them with the means to connect, engage and support others in their efforts to provide a healthy and more equitable environment for the children they educate

## Data Availability

The datasets generated and/or analysed during the current study are not publicly available due to some of the data being potentially identifiable. These data are available from the corresponding author on reasonable request.

## Abbreviations

OSHC: Outside of school hours care
SNAC: Supporting Nutrition for Australian Childcare
SNAC_OSHC: Supporting Nutrition for Australian Childcare in Outside of school hours care
ECEC: Early Childhood Education & Care
